
JOURNAL INFORMATION
==============================
NLM Title Abbreviation: Ga Med Companion
NLM Journal ID: 0011077

The Georgia Medical Companion

Publisher: Biomedical Journal Digitization Project

ARTICLE INFORMATION
==============================
PMCID: PMC8893868
PMID: 36021202
Article version: 1
Subjects: Editorials, Reviews, Etc.

Communications Received

Print publication date: 1872 Dec
Volume: 2
Issue: 12
First page: 775
Last page: 775
License: This work is free of known copyright restrictions. For more information, please see PMC Back Issue Digitization.
License URL: https://creativecommons.org/publicdomain/mark/1.0/

==============================

### COMMUNICATIONS RECEIVED.

Several articles were received too late for this number. They will appear in our next. We again ask our friends to send us articles. We will, in future, give notice of their coming to hand, and what number they will appear in.

---

### ADVERTISING DEPARTMENT.

We are glad to know that our efforts to furnish the readers of the COMPANION a complete advertising reference, has met with their endorsement and approval. We hope to make this department so thorough that the practitioner may consult it at all times when desiring to supply his wants, with absolute certainty, in finding what he needs. None but reliable houses find admission in our pages—knowingly; hence, friends can rely upon getting just what they order.
